# Personality Traits and Psychopathology Over the Course of Six Months of Outpatient Psychotherapy: A Prospective Observational Study

**DOI:** 10.3389/fpsyg.2020.00174

**Published:** 2020-02-07

**Authors:** Michael P. Hengartner, Agnes von Wyl, Barbara Heiniger Haldimann, Misa Yamanaka-Altenstein

**Affiliations:** ^1^Department of Applied Psychology, Zurich University of Applied Sciences, Zurich, Switzerland; ^2^Klaus-Grawe-Institute for Psychological Therapy, Zurich, Switzerland

**Keywords:** psychotherapy, personality, psychopathology, neuroticism, conscientiousness

## Abstract

There is a lack of prospective studies that test pre-specified hypotheses on the role of personality traits in outpatient psychotherapy. A total of 47 patients with a broad range of psychopathological syndromes were enrolled in a prospective 6-month observational study conducted in Zurich, Switzerland. We found evidence for remarkably high differential stability in all Big Five personality traits during the first 6 months of psychotherapy. Mean-level stability was very high in agreeableness and conscientiousness, while modest changes were observed in neuroticism, extraversion and openness. Baseline scores in neuroticism and conscientiousness at the beginning of therapy predicted modest change in self-reported severity of psychopathology over 6 months, but no effect was found in association with therapist-rated functional deficits. We conclude that personality trait levels may change slightly over the course of 6 months of psychotherapy and that both neuroticism and conscientiousness may have weak associations with the self-perceived improvement in psychopathology.

## Introduction

An increasing body of evidence converges on the conclusion that an adaptive personality is a key to well-being, health, social functioning and quality of life (Krueger et al., 2000; Roberts et al., 2007; Hengartner, 2015; Kotov et al., 2017). Personality has likewise been identified as an important determinant of treatment outcome in psychotherapy (Zinbarg et al., 2008; Lengel et al., 2016). However, most findings published thus far are based on exploratory *post hoc* analyses of data that were collected for other principal research questions, which is prone to false-positive findings (Wagenmakers et al., 2012). Research that included more than a one-time personality assessment in order to study dynamic personality-psychopathology covariation over time in naturalistic samples is scarce and longitudinal studies that included patients with other mental disorders than major depression are almost completely lacking (Hengartner and Yamanaka-Altenstein, 2017). This confirmatory longitudinal study in a representative sample of private psychotherapy outpatients was designed to address these gaps in the literature.

## Personality Related to the Outcome of Psychotherapy

Several researchers have suggested that personality traits may be relevant for the conduct of psychotherapy (Miller, 1991; Harkness and Lilienfeld, 1997; Zinbarg et al., 2008). More specifically, it has been shown that high scores in pre-treatment neuroticism may worsen the outcome of depressive disorders (Bush et al., 2004; Spek et al., 2008; Thibodeau et al., 2015); for review see Mulder (2002). There is also evidence that low extraversion and conscientiousness may relate to poorer treatment outcome (Ogrodniczuk et al., 2003; Quilty et al., 2008); for review see Zinbarg et al. (2008). In line with these findings, Barlow et al. (2014) advocated that in persons with emotional disorders, psychological treatment should be targeted directly toward neuroticism. However, there are at least two important gaps in the literature. Firstly, almost all studies focusing on treatment effects of personality traits were based on data derived from randomized controlled trials (RCT) (Bush et al., 2004; Quilty et al., 2008; Spek et al., 2008; Tang et al., 2009). Even though RCT are rightly seen as the gold-standard to evaluate causality due to high internal validity, they typically comprise selective samples that are not representative for the broader help-seeking patient population and they are conducted under artificial laboratory conditions, thus external validity is limited (Westen et al., 2004; Mulder et al., 2018). Secondly, as most trials mainly focused on personality-effects in patients with depressive disorders (Bush et al., 2004; Quilty et al., 2008; Spek et al., 2008; Tang et al., 2009), there is a lack of evidence whether these findings apply to other disorders than depression and to sub-diagnostic psychosocial problems.

## Stability of Personality Over the Course of Psychotherapy

It has been shown that personality trait scores may change in depressed patients during the course of treatment (De Fruyt et al., 2006; Tang et al., 2009; Soskin et al., 2012). Interestingly, evidence from pharmacological and psychotherapeutic trials suggests that personality change is mostly independent of depressive symptoms and placebo response (De Fruyt et al., 2006; Tang et al., 2009; Spittlehouse et al., 2010; Knorr et al., 2012). Personality change could therefore inform a true treatment effect (Costa et al., 2005; Morey et al., 2010), and a recent meta-analytic evaluation concluded that personality traits change significantly during psychotherapeutic intervention (Roberts et al., 2017). This meta-analysis focused on “mean-level stability,” that is, change in mean trait scores in a given sample over time as detailed in De Fruyt et al. (2006). Conversely, another meta-analysis has shown that stability of personality traits is very high over time in clinical samples, suggesting that the impact of psychiatric treatments on personality change might be rather modest (Ferguson, 2010). Importantly, in this meta-analysis, stability of personality traits was calculated as a pre-post correlation coefficient. De Fruyt et al. (2006) referred to this type of stability as “differential stability,” and it measures the trait level of a given individual relative to a broader reference group. Research into the stability of personality traits over the course of psychotherapy should therefore ideally focus on both mean-level stability and differential stability, but this has hardly been done to date.

## The Present Study

In this study we will test bi-directional associations between personality and psychopathology. That is, we will not only focus on the prospective association of pre-treatment personality with the outcome of psychotherapy, but also on the stability and change of personality traits over the course of psychotherapy. These are important issues that are insufficiently addressed in the literature. All hypotheses detailed below were outlined in the pre-registered study protocol (Hengartner and Yamanaka-Altenstein, 2017).

First, we hypothesized that all Big Five traits would show high differential stability (pre-post correlation: *r* > 0.6). Second, all Big Five traits will demonstrate moderate-to-high mean-level stability (mean change: *d* < 0.5). More specifically, for neuroticism we predicted *d* < 0.5 (moderate stability), and for all other traits *d* < 0.2 (high stability). Third, baseline scores in neuroticism and conscientiousness relate strongly to change in psychopathology and functioning (*d* > 0.5). Fourth, mean-level change over time in Big Five traits correlate weakly, if at all, with change in psychopathology and functioning (*r* < 0.3). These hypothesized effect size estimates were derived from the literature reviewed in Hengartner and Yamanaka-Altenstein (2017) and were informed by traditional benchmarks used in clinical psychology to differentiate small from substantially large effect sizes (e.g., references Roberts et al., 2017; Ferguson, 2010). For instance, various authors have suggested that neuroticism and conscientiousness are the most important personality traits related to psychopathology and global functioning (Roberts et al., 2007; Zinbarg et al., 2008; Lengel et al., 2016), therefore we postulated that the association between these two traits and psychopathology/functioning should be at least of medium effect size (i.e., *d* > 0.5).

## Materials and Methods

### Participants and Procedures

All participants were enrolled at the Klaus-Grawe-Institute (KGI) for psychological therapy in Zurich, Switzerland. This is a private outpatient psychotherapy centre offering psychological therapy and counseling according to Grawe’s (2004) framework of general psychology and psychotherapy. It offers a comprehensive application of evidence-based techniques of various psychotherapeutic orientations, but all therapists are trained in and deliver cognitive-behavioral therapy. In total seven different therapists, all women, were involved in the treatment of the included patients. We included all German speaking patients between 18 and 65 years who started psychotherapy at the KGI in Zurich from September 2015 onward and who remained in therapy for at least 6 months. Even though therapy is also offered to English speaking patients, restriction to German speaking patients was made because assessments in English comprise different questionnaires. Assessments were made at the beginning of the therapy (baseline assessment T0) and after 6 months (6-month assessment T1). Data collection lasted from September 2015 (first baseline assessment) to January 2019 (last 6-month assessment). A total of 165 German speaking participants completed the baseline assessment, and of these, 47 patients (28.5%) were still in therapy after 6 months and thus also completed the 6-month assessment. Note that sample attrition is not the result of study discontinuation, but was due the fact that most participants had only a few therapy sessions and thus terminated treatment before the 6-month assessment. Thus, it is not the case that 71.5% of patients were lost to follow-up, but rather that these did not meet inclusion criteria and hence were not eligible for the longitudinal analysis. Based on *a priori* power analysis detailed in Hengartner and Yamanaka-Altenstein (2017), we aimed at 100 6-month assessments. Unfortunately, the participant flow was much lower than initially expected. The end of data collection was expanded twice, but when we were at risk of overrunning our study budget, sampling of baseline data was stopped in August 2018.

This study was pre-registered online using the open science framework^1^ and the study protocol was pre-specified and published online (Hengartner and Yamanaka-Altenstein, 2017). All participants gave their informed consent and the study was approved by the Cantonal Ethics Committee of Zurich (reference number 2015-0601). The project was financially supported by a grant from the OPO Foundation (reference number 2016-0038) granted to principal investigator Dr. Hengartner.

### Instruments and Measures

Normal-range personality was assessed with a German adaptation (Lang et al., 2001) of the well-established Big Five Inventory (BFI) (John et al., 1991). The BFI is a brief self-report inventory capturing the basic structure of personality based on the broad domains of neuroticism, extraversion, openness, conscientiousness and agreeableness. The BFI has been validated in diverse samples across nations, including English, German, Dutch and Chinese, and is considered a reliable and valid short assessment of broad personality traits (John et al., 1991; Lang et al., 2001; Denissen et al., 2008; Carciofo et al., 2016).

Subjective psychopathological symptoms were assessed with the German translation (Franke, 2000) of the Brief Symptom Inventory (BSI) (Derogatis and Melisaratos, 1983). The BSI captures the following nine syndromes: somatization, obsessive-compulsive, interpersonal sensitivity, depression, anxiety, hostility, phobia, paranoia, and psychoticism. Although these scales demonstrated good reliability (Derogatis and Melisaratos, 1983; Franke, 2000), due to rather poor convergent and discriminant validity, it has been suggested to use only a sum of all syndromes (referred to as Global Severity Index, GSI) (Boulet and Boss, 1991).

Global functioning of the patients was rated by the treating therapist with the Global Assessment of Functioning (GAF) scale (American Psychiatric Association [APA], 1994). This measure combines both symptom severity and functional impairment. In addition to the inventories and rating scales detailed above, we also included information from the individual patient chart, such as diagnoses according to ICD-10 code (World Health Organization [WHO], 1992), age, sex, and education level.

### Statistical Analysis

All analyses were conducted with SPSS version 24 for Windows according to the analytic plan pre-specified in the study protocol (Hengartner and Yamanaka-Altenstein, 2017). Mean-level stability was analyzed with paired-samples *T*-tests and differential stability with Pearson correlations between baseline and follow-up scores. The associations between baseline personality trait scores and changes in psychopathology and functioning over time were examined with a series of Generalized Estimating Equations (GEE) (Zeger et al., 1988). These regression models can account for the intra-individual dependency of repeated outcome measures. As detailed in the study protocol (Hengartner and Yamanaka-Altenstein, 2017), the influence of baseline personality on both GAF and BSI-GSI over time was modeled as an interaction term between the personality trait score and the time slope, while adjusting for the main effect of the personality trait and either GAF or BSI-GSI. Because GAF was left-skewed, scores were inverted to transform the scale into a right-skewed distribution. This allowed us to fit these models with Gamma distribution and Power(−1) link-function. In consequence, the inverted GAF score no longer measures functioning, but rather functioning deficits. Finally, the distribution of BSI-GSI scores was half-normal, thus this outcome was modeled with an inverse Gauss distribution and Power(−2) link-function.

## Results

Baseline characteristics of the sample are shown in Table 1. There was a preponderance of women (70.2%) and a high percentage of clients with an university degree (63.6%). The majority did not exhibit any psychiatric diagnosis (59.6%), whereas 25.5% had an anxiety or stress-related disorder (ICD-10 F4) and 12.8% had a depressive disorder (ICD-10 F3). With a mean GAF score of 74.1, the average participant in this sample had only minor symptoms and few functional impairments at the beginning of therapy. Only two participants (4.3%) received concomitant psychopharmacological treatment, one patient for a generalized anxiety disorder and the other for a recurrent depressive disorder (both used antidepressants).

**TABLE 1:** Socio-demographic and clinical characteristics.

**Variable**		**Number (%) or Mean (SD)**
Gender	Woman	33 (70.2)
	Man	14 (29.8)
Age		32.7 (7.4)
Marital status	Married	12 (25.5)
	Unmarried	35 (74.5)
Romantic relationship	In relationship	30 (63.8)
	Single	17 (36.2)
Highest educational degree	Primary school	1 (2.3)
	Secondary school	2 (4.5)
	High school	7 (15.9)
	Higher education	6 (13.6)
	University	28 (63.6)
Psychiatric diagnosis	No diagnosis	28 (59.6)
	Depressive disorder	6 (12.8)
	Anxiety and stress-related disorder	12 (25.5)
	Personality disorder	1 (2.1)
Psychopharmacological treatment	No	45 (95.7)
	Yes	2 (4.3)
GAF score		74.1 (14.0)

Personality trait scores at beginning (T0) and after 6 months of therapy (T1) are depicted in Table 2. Neuroticism scores decreased significantly over time (mean change: −0.193, SD = 0.580, *T* = −2.251, df = 45, *p* = 0.029). Significant increases in mean scores were observed for extraversion (mean change: 0.171, SD = 0.456, *T* = 2.547, df = 45, *p* = 0.014) and openness (mean change: 0.159, SD = 0.312, *T* = 3.445, df = 45, *p* = 0.001), but not for agreeableness (mean change: 0.063, SD = 0.425, *T* = 0.997, df = 45, *p* = 0.324) and conscientiousness (mean change: 0.104, SD = 0.441, *T* = 1.597, df = 45, *p* = 0.117). Accordingly, mean level stability was very high for agreeableness and conscientiousness (*d* = 0.10 and *d* = 0.18, respectively) and somewhat lower for neuroticism (*d* = 0.28), extraversion (*d* = 0.23) and openness (*d* = 0.28). Differential stability was high to very high and ranged from *r* = 0.65 for neuroticism to *r* = 0.85 for openness (all *p* < 0.001).

**TABLE 2:** Big Five trait scores at the beginning (T0) and after 6-months of therapy (T1) and stability over time.

**Persoanlity trait**		**T0**		**T1**	**Stability over time**	
			
	**M (SD)**	**α**	**M (SD)**	**α**	**r**	**d**
Neuroticism	1.96 (0.68)	0.79	1.76 (0.71)	0.86	0.65***	0.28*
Extraversion	2.31 (0.66)	0.81	2.49 (0.80)	0.90	0.82***	0.23*
Openness	2.65 (0.58)	0.80	2.81 (0.56)	0.82	0.85***	0.28**
Agreeableness	2.62 (0.66)	0.80	2.68 (0.61)	0.81	0.78***	0.10
Conscientiousness	2.69 (0.59)	0.80	2.79 (0.57)	0.79	0.71***	0.18

* *p* < 0.05, ** *p* < 0.01, and *** *p* < 0.001.

The results for the association of baseline personality with the outcome of therapy are reported in Table 3. No association between baseline personality scores and change in functional impairment (GAF inverse) over time was observed. However, with respect to changes in subjective severity of psychopathology (BSI-GSI), higher scores in neuroticism were related to lower symptom reduction over time (*b* = −0.334, *p* = 0.012), whereas higher scores in conscientiousness were related to higher symptom reduction over time (*b* = 0.267, *p* = 0.018). The effect sizes corresponded approximately to *d* = 0.45 for neuroticism and to *d* = 0.30 for conscientiousness. Both associations remained almost unaltered and statistically significant when adjusted for the presence of a psychiatric diagnosis in an exploratory *post hoc* analysis.

**TABLE 3:** Personality trait scores at the beginning of therapy (T0) related to change in psychopathology and functional impairment over time (T0–T1).

**Personality trait**	**Psychopathology over time (GSI)**		**Functional impairment over time (GAF)**	
		
	**B (95%-CI)**	**p**	**B (95%-CI)**	**p**
Neuroticism	−0.334 (−0.593; 0.074)	0.012	0.003 (−0.004; 0.010)	0.415
Extraversion	0.122 (−0.119; 0.362)	0.321	0.005 (−0.003; 0.012)	0.680
Openness	−0.013 (−0.583; 0.556)	0.964	0.004 (−0.004; 0.012)	0.305
Agreeableness	−0.072 (−0.352; 0.208)	0.614	0.002 (−0.004; 0.007)	0.588
Conscientiousness	0.267 (0.045; 0.489)	0.018	0.001 (−0.006; 0.007)	0.827

Finally, the results for correlation between change scores of personality and psychopathology are shown in Table 4. Only one significant correlation between change in neuroticism and change in functional impairment (GAF inverse) over time was observed (*r* = 0.33, *p* = 0.028).

**TABLE 4:** Correlation of change in personality with change in psychopathology and functioning.

**Personality trait**	**Psychopathology (GSI)**	**Functional impairment (GAF)**
Neuroticism	0.24 (*p* = 0.106)	0.33 (*p* = 0.028)
Extraversion	−0.22 (*p* = 0.134)	−0.11 (*p* = 0.469)
Openness	0.03 (*p* = 0.847)	−0.10 (*p* = 0.532)
Agreeableness	0.05 (*p* = 0.728)	0.22 (*p* = 0.135)
Conscientiousness	−0.07 (*p* = 0.655)	−0.05 (*p* = 0.743)

## Discussion

The aim of this study was to test pre-registered hypotheses, including an exact specification of the methodological procedure and statistical analysis. Therefore, this research was purely confirmatory according to Wagenmakers et al. (2012). We believe that this is the first original study on the association between personality and psychopathology in psychotherapy clients that was pre-registered online^2^ and pre-specified in an open-access research protocol (Hengartner and Yamanaka-Altenstein, 2017). Moreover, to the best of our knowledge ours is one of the few studies to focus on both differential stability and mean-level stability. Our results show that the differential stability of all Big Five traits was high and that the mean-level stability was moderately high (neuroticism, openness) to very high (agreeableness and conscientiousness). These findings were largely in line with our hypotheses. However, contrary to expectation, baseline neuroticism and conscientiousness did not predict change in therapist-rated global functioning over 6 months of psychotherapy. Moderately weak associations were found between both baseline neuroticism and conscientiousness and change in self-reported severity of psychopathology. Finally, we found a moderate correlation between change in neuroticism and change in functional impairment.

Our findings confirm previous work that concluded that personality traits remain relatively stable over the course of acute psychiatric treatment (which includes both psychotherapy and pharmacotherapy) (De Fruyt et al., 2006), but some trait levels, in particular neuroticism (decrease) and extraversion (increase), appear to change over the course of therapy (Tang et al., 2009). These findings were recently confirmed in a comprehensive meta-analysis by Roberts et al. (2017). That is, concerning trait levels, personality not only develops in the long-term, it may also change weakly, but steadily, in the short-term (Roberts et al., 2006; Roberts, 2018). Conversely, differential stability, that is, the individual standing on a trait relative to others, is remarkably high, as confirmed by a comprehensive meta-analysis by Ferguson (2010). This means for instance, that a person who is relatively neurotic compared to others at the beginning of therapy will largely remain a high-scorer relative to others, even after 6 months of intensive psychotherapy. However, as detailed above, this does not exclude the possibility that the absolute level of neuroticism in fact decreased slightly.

These different aspects of trait stability, and their association with the outcome of psychotherapy, were also discussed by Zinbarg et al. (2008). However, since our study did not include a control group of untreated participants, we cannot exclude that the observed changes in personality traits are due to regression to the mean. That is, our results do not imply that observed changes in personality traits are caused by the psychotherapeutic interventions. Observed changes could also be an artifact due to slightly altered response tendencies (changes in self-perception or awareness) rather than the result of true trait change. Comparing change in self- and observer-ratings could thus be a promising objective in future research, as we still have a poor understanding of how relevant others perceive a patient’s personality change.

We found no evidence that baseline personality trait scores predict change in global functioning over 6 months of therapy as rated by the therapists. This finding was rather surprising, given that several reviews have stressed that in particular neuroticism and conscientiousness are influential for social functioning, general health, and quality of life (Lahey, 2009; Bogg and Roberts, 2013; Hengartner, 2015; Lengel et al., 2016). We found, however, a small-to-medium sized association of neuroticism and conscientiousness with the change of self-reported severity of psychopathology. One possible explanation for why personality appears to predict subjective distress but not therapist-rated impairments would be that patients and therapists have different evaluation criteria (Vazire and Mehl, 2008; Vazire, 2010). Another reason could be that psychotherapy changes a patient’s self-perception, that is, an individual’s characteristic self-defining narrative (McAdams and Pals, 2006; Pasupathi and Wainryb, 2010). Such influences presumably only show in self-reports, but not necessarily in observer-ratings. The significant association between self-rated personality traits and self-rated psychopathology could, at least in part, also be the result of common method bias (Podsakoff et al., 2003).

We would like to highlight some important limitations. First, and perhaps most importantly, the final sample was just half the size we initially planned to collect. The reason for this discrepancy is that when planning the study we considerably overestimated the proportion of treatment beginners who would remain in therapy for at least 6 months. As it turned out, the vast majority of therapies and counseling sessions did not last 6 months. Main reasons included a high percentage of short-time interventions and counseling services, and a rather low proportion of clients with severe mental health problems that required at least 6 months of therapy. Second, we did not include an observer-rating of the Big Five traits, as we did not want to further occupy the patients’ time. Third, although the sample was representative for this private outpatient psychotherapy institute, it is important to acknowledge that most clients were highly educated, of high socio-economic background and most presented with relatively good psychosocial functioning. The study results thus do not necessarily generalize to the broader population of psychotherapy users and to the clients with more severe mental disorders seen in public inpatient psychiatric services. Fourth, since this study did not include a control group, changes observed in the psychotherapy group are not necessarily attributable to therapy effects and thus could also reflect regression to the mean and spontaneous remission (Hengartner, 2019).

Despite these limitations we conclude that personality trait levels may change over the course of 6 months of psychotherapy. However, differential stability, that is, an individual’s standing relative to others, appears to be highly stable. Finally, there was no evidence that baseline personality traits predict changes in global functioning over time, but both neuroticism and conscientiousness moderately predicted changes in subjective symptom severity. These findings indicate that the association between personality traits and psychopathology is rather weak and possibly inflated due to common method bias.

## Data Availability Statement

The datasets generated for this study are freely available via open science framework (https://osf.io/ukbs5/).

## Ethics Statement

The studies involving human participants were reviewed and approved by the Cantonal Ethics Committee of Zurich. The patients/participants provided their written informed consent to participate in this study.

## Author Contributions

MH was the principal investigator, designed the study, analyzed the data, wrote, and revised the manuscript. MY-A participated in design and conduct of the study, interpretation of the data, writing, and revision of the manuscript. AW and BH participated in writing and revision of the manuscript.

## Conflict of Interest

The authors declare that the research was conducted in the absence of any commercial or financial relationships that could be construed as a potential conflict of interest.

## Footnotes

1 https://osf.io/ukbs5/

2 https://osf.io/ukbs5/
